# MEANS: python package for Moment Expansion Approximation, iNference and Simulation

**DOI:** 10.1093/bioinformatics/btw229

**Published:** 2016-05-05

**Authors:** Sisi Fan, Quentin Geissmann, Eszter Lakatos, Saulius Lukauskas, Angelique Ale, Ann C. Babtie, Paul D. W. Kirk, Michael P. H. Stumpf

**Affiliations:** ^1^Centre for Integrative Systems Biology and Bioinformatics, Department of Life Sciences, Imperial College London, London SW7 2AZ, UK; ^2^MRC Biostatistics Unit, Cambridge CB2 0SR, UK

## Abstract

**Motivation**: Many biochemical systems require stochastic descriptions. Unfortunately these can only be solved for the simplest cases and their direct simulation can become prohibitively expensive, precluding thorough analysis. As an alternative, moment closure approximation methods generate equations for the time-evolution of the system’s moments and apply a closure ansatz to obtain a closed set of differential equations; that can become the basis for the deterministic analysis of the moments of the outputs of stochastic systems.

**Results**: We present a free, user-friendly tool implementing an efficient moment expansion approximation with parametric closures that integrates well with the IPython interactive environment. Our package enables the analysis of complex stochastic systems without any constraints on the number of species and moments studied and the type of rate laws in the system. In addition to the approximation method our package provides numerous tools to help non-expert users in stochastic analysis.

**Availability and implementation:** https://github.com/theosysbio/means

**Contacts:** m.stumpf@imperial.ac.uk or e.lakatos13@imperial.ac.uk

**Supplementary information:** Supplementary data are available at *Bioinformatics* online.

## 1 Introduction

Inherent stochasticity of elementary reactions affects many biochemical processes. Analytic solutions of stochastic systems are rarely available and simulations (Gillespie, 1977) can come with high computational costs making the study of such systems implausible. Numerous approximation methods have been developed recently to lighten the burden. A popular approach is to approximate the whole system by following the moments of the temporally evolving probability distribution, such as in the Moment Expansion Approximation (MEA) (Ale *et al.*, 2013). However, for nonlinear systems the equation set arising from such methods is in principle infinite. Moment closure formulae address this problem by offering a substitute expression of moments over a certain order, based on information contained in the remaining moments and prior assumptions on the system (Gillespie, 2009). For a more detailed literature review on this technique see Lakatos *et al.* (2015).

Here we present MEANS, a Python package for ***M****oment* ***E****xpansion* ***A****pproximation, i****N****ference and* ***S****imulation*. Many existing packages with a similar function restrict the calculation to polynomial reaction rates; besides, an analysis based on them might require expensive softwares or computational skills not generally available to all potential users (Azunre *et al.*, 2011; Schnoerr *et al.*, 2015; see also http://www.ece.ucsb.edu/hespanha). Our package on the other hand, provides an automated moment equation generation method, suitable for arbitrary rate laws; together with the choice of distribution-based closure formulae, including a multivariate gamma not available in other packages. Furthermore, the package is complemented with an implementation of exact stochastic simulation, as well as support for different ODE solvers and sensitivity analysis, a parameter estimation tool and specific functions for the visualization of results.

## 2 Approach

For a thorough description of the moment expansion and closure algorithms we refer the user to Ale *et al.* (2013) and Lakatos *et al.* (2015). In brief, given a stochastic system of *N* species by its stoichiometry matrix (*S*) and reaction propensities (*a*), the evolution of the species’ probability distribution *P*(*x*, *t*) is described in terms of its moments, which can be evaluated through its moment generating function (wrt θ),

(1) $$\frac{dm}{dt}=\sum_{l}\left[\left(e^{\theta S_{l,:}}-1)\sum_{x}e^{\theta x}P(x)a_{l}(x\right)\right].$$

The expressions thus obtained are then evaluated through two subsequent Taylor expansions. Two steps are used to derive a closed ODE system: first, for an approximation based on *m*th order moments, the expansion is stopped at order (m+1); then moment closure formulae (*mc^k^*) are used to substitute the highest order terms with expressions of means (μ) and (co)variances (Σ)

(2) $$\frac{d}{dt}E({\hat{x}}^{m})=f(\mu ,\ldots ,E({\hat{x}}^{m}),mc^{\left(m+1\right)}(\mu ,\Sigma )).$$

## 3 Methods

MEANS is a Python package, primarily developed to be used interactively in the IPython/Jupyter notebook framework. Pre-requisites and installation instructions are listed in the accompanying documentation. MEANS enables the definition and exact simulation of stochastic models; moment-based approximations for arbitrary stochastic system and closure order; numerical solution, sensitivity analysis and parameter inference of ODE systems generated by the approximation or defined by the user; and customizable visualization for the output. Detailed tutorial notebooks demonstrating the functionalities of the package are available from the tutorial directory.

*Input:* The stochastic system can be supplied by manual input of a symbolic representation of the variables and reactions, all supported by the SymPy library (SymPy Development Team, 2014); this is then converted into a specific model object. There is also a parser enabling model definition from SBML-files. Furthermore, four predefined models are available from the **examples** package. Our package also offers a set of routines for serialization and deserialization of the MEANS-specific objects to and from files, therefore previously defined models are easily saved and loaded.

*Approximation and closure:* The MEA method can be called through the **mea_approximation** function, which requires two arguments: a MEANS model object and the closure order. The default closure method is *scalar* (truncation); distribution-based methods can be used by setting the **closure** argument of the function. MEANS supports *normal, lognormal* and *gamma* closures, the latter based on a new definition of multivariate gamma distribution designed to fit general biochemical networks (Lakatos *et al.*, 2015). When univariate distributions are desired, the parameter **multivariate = False** can be passed to the closure function. We can also generate Linear Noise Approximation expressions by calling the function **lna_approximation** where the model is the single argument.

*Simulation:* MEANS uses Assimulo (Andersson *et al.*, 2015) as the ODE solver back-end, and supports all solvers and settings implemented in that package, which can be accessed via the optional argument **solver**. The default value is *ode15s*, which corresponds to the MATLAB function with the same name. The simulation returns a collection of Trajectory objects, each containing a list of time-points, values and a description of the moment. Stochastic systems can be also directly simulated in MEANS by providing the model and the number of simulations as input.

Furthermore, parameter sensitivities can be computed while performing the ODE simulations using the built-in support of CVODE solvers (Hindmarsh *et al.*, 2005). Sensitivity analysis returns a collection of trajectory objects with additional .**sensitivity_data** attribute.

*Inference:* Parameter estimation is also available in MEANS; for this function the observed data can be entered manually or read from a file; and then converted into a Trajectory object with appropriate descriptors so that means and higher moments can be matched. Variable parameters are defined by a dictionary of the symbolic names and corresponding ranges of allowed values; range *None* indicates unbounded parameters, and all unspecified parameters are assumed constant. Starting values for the parameters can be user-defined or generated automatically by optimizing runs from random starting positions sampled from a defined range; we advise the latter to lower the chance of finding local minima.

**Fig. 1.**
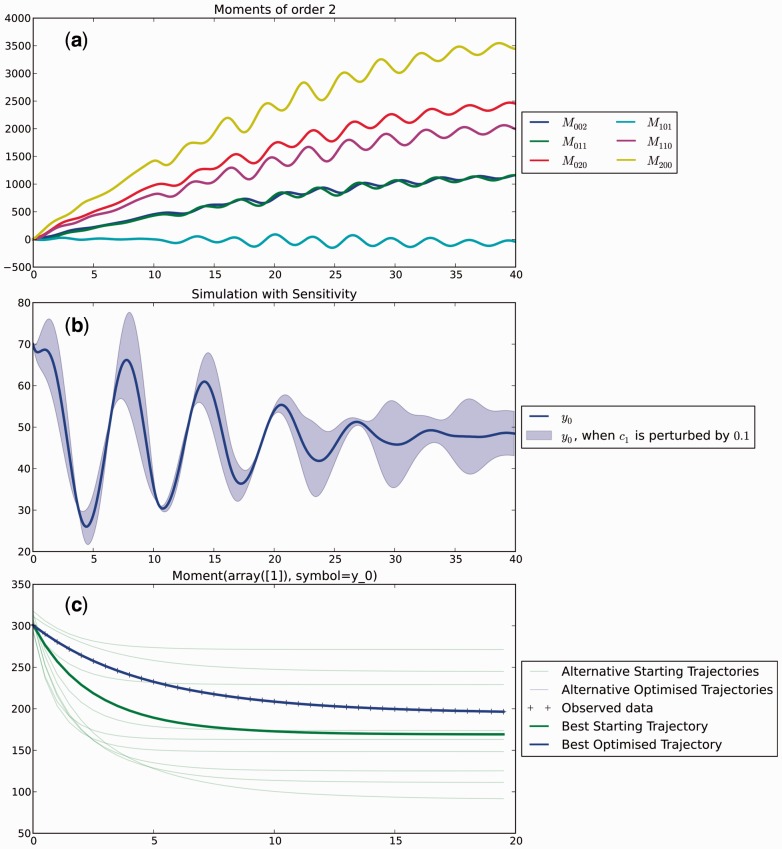
Default graphical output of MEANS. Labels are automatically generated. (**a**) Second order moments. (**b**) Outcome of sensitivity analysis: original trajectory with region covered if a single parameter is perturbed. (**c**) Result of inference with restarts: light lines show starting trajectories considered, dark lines show the best starting position and the final fit (Color version of this figure is available at *Bioinformatics* online.)

*Visualization:* Trajectory objects can be visualized in the notebook, see Figure 1(a). A customizable .**plot()** method allows the use of all options of the standard matplotlib plot function. Sensitivity behaviour can be illustrated using .**plot_perturbations()**, which visualizes the result of perturbing a single parameter with a specified delta value, Figure 1(b). Inferred trajectories can also be visualized using the result object’s .**plot()** method, cf. Figure 1(c). MEANS also has functions to plot the distance landscape and intermediate solutions in order to aid the analysis.

## 4 Summary

We present MEANS, a Python package, for the approximation of the time-evolution of stochastic biochemical networks using moment expansion with moment closure formulae based on multivariate normal, lognormal and newly defined gamma distributions. Besides the derivation of moment equations, MEANS also offers several methods to simulate stochastic models, as well as tools for sensitivity analysis and parameter inference.

## Supplementary Material

Supplementary Data
