# Supplementary material for: MEANS: python package for Moment Expansion Approximation, iNference and Simulation
Source: Bioinformatics. 2016 May 5;32(18):2863–5. doi: 10.1093/bioinformatics/btw229 (PMC5018365; doi:10.1093/bioinformatics/btw229)
Supplement: Supplementary Data [file supp_32_18_2863__index.html]

MEANS: python package for Moment Expansion Approximation, iNference and Simulation — MEANS: python package for Moment Expansion Approximation, iNference and Simulation — Supplementary Data 

# MEANS: python package for Moment Expansion Approximation, iNference and Simulation

## Supplementary Data

files

- Supplementary Data - pdf file
