# Supplementary material for: MEANS: python package for Moment Expansion Approximation, iNference and Simulation
Source: Bioinformatics. 2016 May 5;32(18):2863–5. doi: 10.1093/bioinformatics/btw229 (PMC5018365; doi:10.1093/bioinformatics/btw229)
Supplement: Supplementary Data [file supp_btw229_means_applnote_si.pdf]

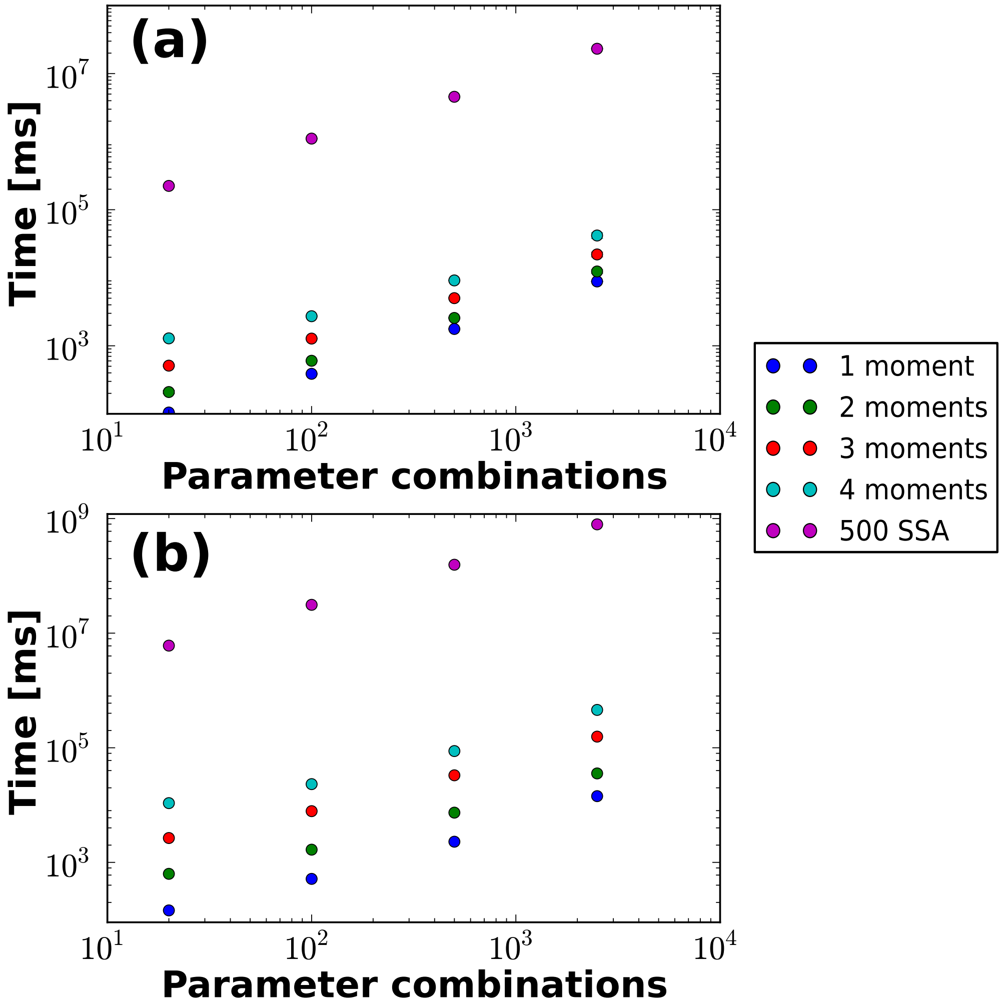

Figure 1: Computational time required to simulate the (**a**) Michaelis-Menten (**b**) p53-Mdm2 oscillatory system in a parameter scan task, using MEANS with different closure orders and trajectories averaged from 500 runs of Gillespie's Stochastic Simulation Algorithm (SSA). Parameters to be scanned are set deterministically to be the same in all cases so that variations of running time arising from different reaction rates are accounted for. MEANS data points also contain the time to perform the moment closure to generate the underlying ODE system. Note that the average obtained from 500 SSA runs is still affected by noise and in practice an ensemble of more realisations should be used, especially for predicting higher order moments.
